# Enrichment by hybridisation of long DNA fragments for Nanopore sequencing

**DOI:** 10.1099/mgen.0.000087

**Published:** 2016-09-20

**Authors:** Sabine E. Eckert, Jackie Z.-M. Chan, Darren Houniet, the PATHSEEK consortium,, Judy Breuer, Graham Speight

**Affiliations:** ^1^​ Oxford Gene Technology, Begbroke Science Park, Begbroke Hill, Woodstock Road, Begbroke, Oxfordshire OX5 1PF, UK; ^2^​ a list of participants can be found in the Acknowledgements; ^3^​ UCL Division of Infection & Immunity, Cruciform Building, Gower Street, University College London, London WC1E 6BT, UK

**Keywords:** enrichment by hybridisation, human Herpes virus/cytomegalovirus, influenza A, *Mycobacterium tuberculosis*, nanopore sequencing

## Abstract

Enrichment of DNA by hybridisation is an important tool which enables users to gather target-focused next-generation sequence data in an economical fashion. Current in-solution methods capture short fragments of around 200–300 nt, potentially missing key structural information such as recombination or translocations often found in viral or bacterial pathogens. The increasing use of long-read third-generation sequencers requires methods and protocols to be adapted for their specific requirements. Here, we present a variation of the traditional bait–capture approach which can selectively enrich large fragments of DNA or cDNA from specific bacterial and viral pathogens, for sequencing on long-read sequencers. We enriched cDNA from cultured influenza virus A, human cytomegalovirus (HCMV) and genomic DNA from two strains of *Mycobacterium tuberculosis* (*M. tb*) from a background of cell line or spiked human DNA. We sequenced the enriched samples on the Oxford Nanopore MinION™ and the Illumina MiSeq platform and present an evaluation of the method, together with analysis of the sequence data. We found that unenriched influenza A and HCMV samples had no reads matching the target organism due to the high background of DNA from the cell line used to culture the pathogen. In contrast, enriched samples sequenced on the MinION™ platform had 57 % and 99 % best-quality on-target reads respectively.

## Data Summary

The raw datasets from Nanopore and Illumina reads generated in this study were deposited in the European Nucleotide Archive: Study PRJEB12651; http://www.ebi.ac.uk/ena/data/view/PRJEB12651.

## Impact Statement

Our work describes a method for the selective enrichment of known viral or bacterial pathogen DNA from a background of host DNA for sequencing on the Oxford Nanopore MinION™ long-read sequencer. We developed a protocol for enriching large DNA fragments (>1 kb) by in-solution hybridisation, as contrasted to short fragments (200–300 bp) used for second-generation sequencing. In this proof-of-principle experiment, we enriched long DNA fragments of influenza virus A, human cytomegalovirus and *Mycobacterium tuberculosis* from their culture cell line or from a laboratory-made mixture of bacterial and human genomic DNA. We believe our method and evaluation of the results will be of interest to the growing group of users of long-read sequencers (Oxford Nanopore, Pacific Biosciences). For example, this method could be used in the pathogen field for the whole-genome sequencing of small target organisms in mixed/clinical samples and in the identification of structural variants such as translocations in small or large genomes.

## Introduction

While the cost of next-generation sequencing has been falling continuously in recent years, the enrichment of specific DNA regions or whole genomes from microorganisms allows the multiplexing of several samples per run whilst maintaining a high depth of coverage over the regions of interest. The capture of viral and bacterial organisms from mixed samples by in-solution bait hybridisation, followed by high-throughput sequencing, is advantageous for the evaluation of variant frequency and deconvolution of PCR duplicates, compared with the sequencing of PCR-generated amplicons (Samorodnitsky *et al.*, 2015). This enrichment method can be used in a clinical setting to aid and refine timely diagnosis (Wlodarska *et al.*, 2015), for example from extensively or totally drug-resistant pathogens in a time of antibiotic overuse (Carlet, 2015). Data from whole-genome sequencing provides a wealth of information such as identification of resistance markers carried by the infecting agent(s), allowing for rapid, targeted and personalised treatment. Previous studies have shown that it is possible to bypass the traditional culture-based diagnosis and obtain information by sequencing metagenomic samples, but the throughput is low and the method prohibitively costly for routine use (e.g. Doughty *et al.*, 2014, Loman *et al.*, 2013). A potentially disruptive diagnostic platform to sequence enriched bacterial and viral pathogens directly from clinical samples has been previously described by Brown *et al.* (2015) and Christiansen *et al.* (2014). This approach employs custom baits to capture genomic material from the target organisms, thereby reducing the amount of human and commensal DNA in the clinical samples and allowing greater throughput of samples. However, this method is optimised for short-read sequencers such as the Illumina MiSeq and the Ion PGM, and is unsuitable for long-read sequencers. Information from long-read platforms could be used, for example, to resolve highly repetitive regions such as those found in cytomegalovirus (Masse *et al.*, 1992), detect large structural variations (Jiang *et al.*, 2015) or provide evidence of recombination events such as those seen in *Chlamydia trachomatis* (Joseph & Read, 2012). Enrichment of specific genomic fragments by PCR-generated baits for sequencing on an Oxford Nanopore MinION™ sequencer was demonstrated by Karamitros & Magiorkinis (2015).

Here, we present an adaptation of the method used by Brown *et al.* (2015) and Christiansen *et al.* (2014), enrichment of DNA fragments of between 1 and 15 kb for sequencing on long-read platforms. We joined the Oxford Nanopore Technologies (ONT) MinION™ Access Program to assess the suitability of this platform used in combination with the targeted enrichment method. We compared sequence data from unenriched and enriched cultured influenza virus A and HCMV samples, run on the MinION™ and Illumina MiSeq platforms. We also mixed cultured *Mycobacterium tuberculosis* (*M. tb*) genomic DNA from two different strains with human DNA to evaluate the efficiency of enrichment by hybridisation for longer bacterial DNA fragments. We found that the long genomic fragments were readily purified from a background of the cell line used for producing the viruses, or, in case of *M. tb*, mixed with human DNA.

## Methods

### Samples.

Mycobacterial genomic DNA from strain H37Rv was a kind gift from A. Brown, L. J. Schreuder and T. Parish (Barts and The London School of Medicine and Dentistry, Queen Mary University of London, London, UK), and the extensively drug-resistant clinical *M. tb* strain C from P. Butcher and J. Dhillon (Institute of Infection and Immunity, St. George’s Hospital, University of London, UK) (Witney *et al.*, 2015). To test target enrichment, *M. tb* DNA was mixed with human genomic DNA (Male, #G1471, Promega), to 10 % (450 ng human DNA, 50 ng *M. tb* DNA) or 90 % (450 ng *M. tb*, 50 ng human) prior to processing.

RNA from influenza virus strain A A/PR/8/34 H1N1, lot 1115 (1.78×10^13^ TCID_50 _ml^−1^), grown in the MDCK Cocker Spaniel kidney cell line, was obtained from the Public Health England Culture Collection (#0111041v, Porton Down, UK), and reverse transcribed with NEBNext RNA First Strand Synthesis Module #E7525 and NEBNext mRNA Second Strand Synthesis Module #E6111 (New England Biolabs) according to the manufacturer’s instructions. The ds cDNA was cleaned up with DNA Clean & Concentrator columns (#D4013, Zymo Research).

DNA from HCMV strain Merlin grown in fibroblast cell culture (6.75×10^6^ copies µl^−1^, determined by qPCR) was a kind gift of R. Milne at the Department for Virology, UCL Medical School, Royal Free Campus, London, UK.

### Sample preparation and long-fragment hybridisation.

**Fig. 1.**
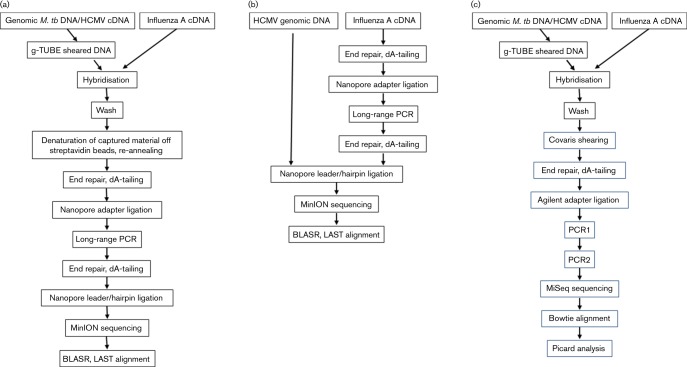
Workflow for hybridisation and sequencing of long-fragment-enriched pathogen DNA. (a) Enrichment and library preparation for the Oxford Nanopore MinION™ sequencer. (b) non-enriched Nanopore libraries, (c) library preparation for long-fragment-enriched Illumina control experiments.

The different workflows for this study are outlined in Fig. 1. HCMV (500 ng, 6.7×10^7^ copies) and* M. tb* samples (500 ng) were diluted in TE to an end volume of 80 µl, and sheared in Covaris g-TUBEs (#520079, Covaris) with two passages at 7200 r.p.m./4200 ***g*** for 1 min in a desktop centrifuge (#5242, Eppendorf). The HCMV genomic DNA was subjected to PreCR (#M0309, New England Biolabs,) enzymatic repair according to the manufacturer's recommendations after shearing (Table 1). Influenza virus A samples were not sheared since the cDNA fragments were size-compatible with Nanopore sequencing. The equivalent of 1×10^12^ TCID_50_ was used for the library preparation from the enriched influenza virus A cDNA.

Concentrations and fragment sizes were determined with a Qubit fluorometer (dsDNA BR Assay Kit #Q32850, Life Technologies), and Agilent Tape Station (Genomic DNA ScreenTape #5067–5365 and Genomic DNA Reagents #5067–5366; High Sensitivity RNA ScreenTape #5067–5579, High Sensitivity RNA ScreenTape Sample Buffer #5067–5580, High Sensitivity RNA ScreenTape Ladder #5067–5581; High Sensitivity D1000 ScreenTape #5067–5584, High Sensitivity D1000 Reagents #5067–5585, Agilent) according to the manufacturers' instructions throughout the experiment.

Biotinylated custom RNA baits for the target organisms influenza virus A (49190 baits), HCMV (33809 baits) and *M. tb* (224612 baits) were designed with an in-house Perl script (Depledge *et al.*, 2011), using a database of 4968 H1N1 and 2966 H3N2 influenza virus A genomes, 115 partial and complete HCMV genomes and the *M. tb* strain H37Rv reference genome (NC_018143.2), respectively, and manufactured by Agilent. Sheared genomic DNA (HCMV, *M. tb*) and cDNA (influenza virus A) samples were hybridised and captured according to the SureSelect^XT^ Target Enrichment for Illumina Paired-End Multiplexed Sequencing protocol (Version B.1, 2014, 16 h hybridisation). Following capture, samples were heated to 95 °C for 3 min, and cooled to 35 °C (ramp: 4 °C min^−1^) to release the target fragments from the baits bound to streptavidin beads.

Half of each hybridised sample was used for Nanopore library preparation with ONT kit versions SQK-MAP003 for *M. tb* strain H37Rv and SQK-MAP004 for HCMV, influenza virus A, and *M. tb* strain C. The remainder was used for the generation of Illumina-compatible libraries.

### Nanopore library preparation, sequencing and analysis.

End repair and dA-tailing of all samples were performed with enzymes from the SureSelect^XT^ kit (#5500–0075, Agilent) and adaptors and primers from the ONT SQK-MAP003 or SQK-MAP004 library preparation kits as specified by the manufacturers. Following AMPure XP bead purification (#A63880, Beckman Coulter), the dA-tailed samples were ligated to adaptors (ONT) for 15 min at room temperature. They were cleaned up with AMPure XP beads and eluted in H_2_O. The ligated DNA was amplified using Long Amp Taq 2x Master mix (#M0287, NEB) and ONT PCR primers with the following program: 95 °C 3 min; 15–18 cycles of 95 °C 15 sec, 62 °C 15 sec, 65 °C 10 min; 65 °C 20 min; 4 °C hold.

A second round of end repair and dA-tailing was performed on 500 ng of enriched, amplified PCR product using SureSelect^XT^ reagents as described above, but without purification after dA-tailing. Instead, leader/hairpin ligation and sample clean-up were performed according to the ONT protocols for kit SQK-MAP003 (used in the *M. tb* strain H37Rv experiments only) or SQK-MAP004. In detail, dA-tailed sample, blunt/TA ligase master mix (#M0367, NEB), tethered adapter mix and hairpin adapters (ONT) were incubated for 10 min at room temperature in protein LoBind tubes (#0030108116, Eppendorf) for ligation. Libraries processed according to the ONT SQK-MAP003 protocol were cleaned up with AMPure XP beads; those made according to the SQK-MAP004 method were purified using Dynabeads for His-Tag isolation and pulldown (#10103D, Life Technologies) (Fig. 1a). Libraries were eluted from the beads by incubation for 10 min at room temperature in elution buffer (ONT). Library concentrations were typically 2–10 ng µl^−1^, as assessed by Qubit fluorometer.

The influenza virus A control sample that did not undergo hybridisation (75 ng, the equivalent of 2.7×10^11^ TCID_50_) was end-repaired, dA-tailed and amplified with Long Amp Taq polymerase as described above. Samples (500 ng) of this PCR product were processed as recommended in the ONT Genomic DNA sequencing protocol SQK-MAP004. For the non-hybridised HCMV sample, 500 ng (4.2×10^7^ copies) were used directly for Nanopore library preparation (SQK-MAP004) without amplification as enough material was available to proceed directly to sequencing (Fig. 1b).

Before each MinION™ run, flowcells were quality-tested with the script MAP_Platform_QC (MinKnow software version 0.46.2.8 to 0.49.2.9), then loaded with 12–60 ng of prepared library, library fuel mix and EP buffer (ONT) as per the manufacturer’s instructions, and run with script MAP_48 Hr_Sequencing_Run, for an average of 26 h.

Reads were analysed by the Metrichor 2D basecalling (versions 2.19 to 2.29) cloud-based platform, and the resulting fast5 files (‘pass’ quality, both strands read while passing through the nanopore, resulting in higher confidence; and ‘fail’, where only one strand is read) converted to fasta format with Poretools (Loman & Quinlan, 2014). BLASR (Chaisson & Tesler, 2012) and LAST (Kiełbasa *et al*., 2011) were used to align reads to the pathogen reference sequences (HCMV herpesvirus HHV-5 GU179001.1, *M. tb* strain H37Rv NC_018143.2, and influenza virus A strain H1N1, A/Puerto Rico/8/1934). Command lines were: ‘./blasr input.fa reference.fa -sam -out output.sam’, ‘samtools view -bS output.sam > output.bam’ for BLASR and ‘lastdb index_input input.fasta’, ‘lastal index_input reference.fa -r1 -a1 -b1 > output.maf’, ‘maf-convert -n sam < output.maf > output.sam’ for LAST, respectively. Files were further tested with both aligners against background human (Human_g1k_v37, www.1000genomes.org) or dog (Ensembl *CanFam3.1* GCA_000002285.2; NC_006583.3) sequences and the ONT adapters used for PCR.

### Illumina library preparation from long, hybridisation-enriched fragments.

For the generation of Illumina libraries, half of each hybridised sample (*M. tb* strain H37Rv, *M. tb* strain C, HCMV and influenza virus A) were sheared with a Covaris AFA instrument (Covaris) to 200 nt fragment size and converted into Illumina-compatible libraries (Fig. 1c) using Agilent reagents and SureSelect^XT^ protocol steps as before. Briefly, samples were end-repaired, dA-tailed, had adapters ligated and were PCR-amplified (six cycles) as described in the protocol. Following sample purification, the PCR products were re-amplified using post-capture indexed PCR2 primers for a further 15 cycles. Sequencing (2×300 nt read length) was performed on an Illumina MiSeq instrument with paired-end 600V3 kits (#MS-102-3003) with automatic adapter trimming. Results from the Illumina MiSeq runs were aligned to the respective references with Bowtie version 1.1.1 (http://bowtie-bio.sourceforge.net/index.shtml). Additional alignment metrics from the bam files were obtained using the Picard CollectMultipleMetrics (http://broadinstitute.github.io/picard/) tool, which generates metrics such as percentage of reads aligned to a given target as well as coverage data.

## Results

### Comparison of Nanopore library size and read length

Table 1 shows the peak sizes of the DNA samples after shearing, as determined on an Agilent Tape Station. The size distribution of the influenza virus A RNA and cDNA prior to processing, showed distinct peaks at 160 nt, 320 nt, 500 nt, 670 nt, 900 nt, 1.2 kb, 3 kb, (Fig. S1a, b, available in the online Supplementary Material), with fragments up to 15 kb. These were presumably short fragments of the eight influenza virus A segments NC_002016 to NC_002023, and residual dog cell line DNA. The size of fragments pre- and post-reverse transcription were broadly similar (Fig. S1). Due to the shortness of the fragments, influenza virus A samples were not sheared.

**Table 1.** Average size of DNA fragments at various stages during the protocol The table shows the modal fragment size post-shear and post-PCR, and mean Nanopore read length (‘pass’, and ‘fail’ read quality) with standard deviations (sd), of the samples used in this study.

Samples	Average (modal) size of DNA fragments		Average (mean) read length	
	Post-shear (nt)	Post-PCR (nt)	‘Pass’ reads [nt (sd)]	‘Fail’ reads [nt (sd)]
Influenza virus A non-enriched*	160, 320, 500, 670, 900, 1250, 3000+*	99–4000	1598 (1191)	805 (946)
Influenza virus A enriched*	160, 320, 500, 670, 900, 1250, 3000+*	370–4000	773 (683)	533 (733)
HCMV non-enriched	12 500†	–‡	3176 (2291)	487 (1203)
HCMV enriched	12 500	1587, 5640	1528 (975)	1083 (1099)
*M. tb* H37Rv	13 800	2000–7000	2402 (1865)	757 (1855)
*M. tb* strain C	15 000	1500	759 (355)	596 (713)

*Influenza virus A samples were not sheared.

†HCMV fragment size after shearing and PreCR.

‡The non-enriched HCMV sample was not PCR-amplified as enough material was available to proceed directly to sequencing.

The HCMV sample (g-TUBE-sheared and PreCR-treated) had a tight range of fragment sizes of around 12.8 kb. After PCR amplification, a broad range of fragment sizes both within and between individual reactions were observed. In general, the products were about half the size of the original DNA before hybridisation, ranging between 1.6 kb and 5.6 kb. One exception was strain *M. tb* C, which had shorter (median size 1.5 kb) PCR products.

The Nanopore reads (Table 1) were similarly variable in length, reflecting the input material, as indicated by the standard deviations in Table 1. Sequenced reads were shorter on average than the PCR products, but with a wide range. Reads classified as ‘pass’ quality by the Metrichor platform were longer than ‘fail’ quality reads. Non-hybridised samples had longer read lengths than enriched samples, either due to DNA damage during the hybridisation and wash processes, or preferential amplification of shorter fragments during PCR.

### Comparison of BLASR and LAST aligners

We used BLASR (Chaisson & Tesler, 2012) and LAST (Kiełbasa *et al*., 2011), with the settings used in Quick *et al.* (2014) for the alignment of Nanopore reads to their respective references (pathogen and human/dog cell line). Table 2 shows statistics for the similarities to the target references obtained with the two aligners. We found that BLASR alignment of reads showed slightly higher identity to the references, shorter aligned regions and lower standard deviation. The LAST aligner produced longer alignments with lower identity and higher standard deviation. This is similar to the observations of Kilianski *et al.* (2015). A percentage of reads (10–35 %) aligned to the reference by LAST are not aligned by BLASR, and vice versa, indicating that neither aligner works optimally for aligning Nanopore reads to the reference.

**Table 2.** Mean similarity and length (with standard deviations, sd) of Nanopore reads aligned to the pathogen targets using BLASR and LAST

Sample	BLASR alignment		LAST alignment	
	Mean similarity of reads to target [% (sd)]	Mean length of alignment [nt (sd)]	Mean similarity of reads to target [% (sd)]	Mean length of alignment [nt (sd)]
Influenza virus A enriched	79 (6.4)	201 (121)	74.8 (6.8)	314 (136)
HCMV enriched	76.8 (6.8)	946 (909)	72.1 (7.9)	1413 (986)
*M. tb* H37Rv enriched	76.9 (6.5)	949 (1022)	69.2 (7.6)	1667 (1154)
*M. tb* strain C enriched	79.4 (6.7)	287 (241)	70.7 (7.6)	523 (744)

### Comparison of enriched and non-enriched Nanopore libraries

A total of 13 nanopore sequencing runs were included in our datasets. The average starting pore count per flowcell was 215. Most ‘pass’ quality reads aligned to either the target organism or the respective cell line, whereas most ‘fail’ quality reads did not match to target, cell line (Table 3) or sequences in the PubMed Nucleotide database (November 2015). This has been reported elsewhere (e.g. Greninger *et al.*, 2015; Kilianski *et al.*, 2015). Regions of alignment were shorter than read length, possibly due to regional increase of the error rates within reads.

**Table 3.** Percentages of Nanopore reads aligned to target pathogen and cell line/human DNA in the samples prepared for this study Alignment statistics are the combined results from BLASR and LAST.

Sample	Reads aligned to target pathogen			Reads aligned to cell line/human DNA		
	Percentage of total reads	Percentage ‘pass’ reads	Percentage ‘fail’ reads	Percentage of total reads	Percentage ‘pass’ reads	Percentage ‘fail’ reads
Influenza virus A non-enriched	0.0	0.0	0.0	29.9	98.9	25.1
Influenza virus A enriched	10.9	57.2	9.5	3.7	28.4	2.9
HCMV non-enriched	0.2	0.0	1.0	10.9	100.0	7.2
HCMV enriched	45.5	98.7	35.0	1.1	1.3	1.0
10 %* M. tb* H37Rv enriched	7.3	32.8	3.9	11.4	46.6	6.6
90 %* M. tb* H37Rv enriched	3.4	5.9	1.7	10.5	12.6	9.2
10 %* M. tb* strain C enriched	0.8	5.9	0.8	8.2	23.5	8.2
90 %* M. tb* strain C enriched	4.4	88.1	3.9	5.2	10.3	5.2

**Fig. 2.**
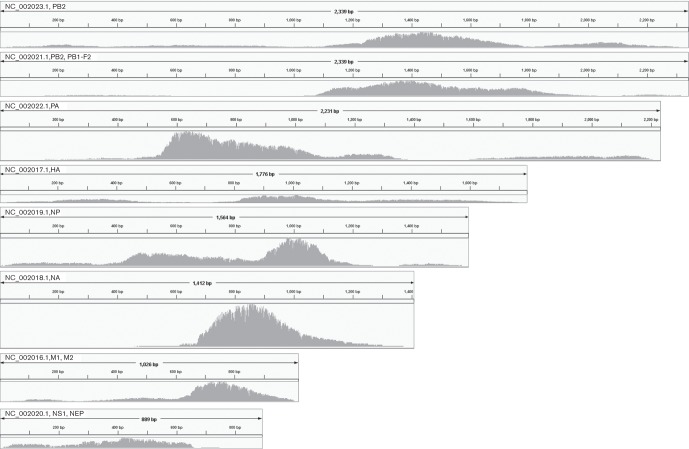
Coverage profile of Nanopore reads from enriched influenza virus A cDNA, aligned to reference H1N1 with BLASR, coverage visualized in the Integrated Genome Viewer (IGV, Robinson *et al*., 2011; Thorvaldsdóttir *et al*., 2013). Maximum read depths for the fragments according to IGV are: 139 (NC_002023.1), 139 (NC_002021.1), 225 (NC_002022.1), 51 (NC_002017.1), 219 (NC_002019.1), 1589 (NC_002018.1), 185 (NC_002016.1), 16 (NC_002020.1).

Analysis of the 42 261 reads obtained from one non-enriched, PCR-amplified influenza virus A cDNA library run on the Nanopore MinION^TM^ found 98.9 % ‘pass’ and 25.1 % ‘fail’ reads aligned to the MDCK dog cell line used for cultivation of the virus, whilst only one read aligned to the influenza virus A reference H1N1. After hybridisation and amplification, 57.2 % of ‘pass’ and 9.5 % of ‘fail’ reads (34 211 reads in total) from one Nanopore run could be aligned to influenza virus A. This amounts to an average read depth of the influenza virus A genome of 62.9×. Fig. 2 shows uneven distribution of reads per fragment, with distinct peaks of increased coverage. This probably reflects the size distribution of the input RNA (Fig. S1a) rather than effects of reverse transcription, hybridisation or PCR bias. The frequency of cell line reads in influenza virus A-enriched samples dropped to 28.4 % (‘pass’) and 2.9 % (‘fail’) (Table 3).

**Fig. 3.**
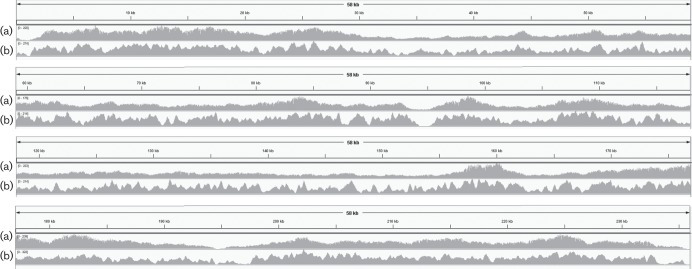
Nanopore reads of HCMV (a), aligned with BLASR to strain HHV-5, coverage visualized with IGV. Maximum read depth 239×. Panel (b), shows an Illumina run generated from a long-fragment enrichment, downsampled to similar coverage of 200–300×.

The unenriched HCMV library (a total of 432 reads from one flowcell) produced four reads (0.2 % of total) matching the HCMV reference HHV-5, while 47 reads (10.9 % of total) matched the human_g1k_v37 reference. After enrichment of the DNA with the HCMV-specific bait set, we obtained 37 589 reads from three runs, with almost all (98.7 %) ‘pass’ reads and 35 % of ‘fail’ reads aligning to the HCMV reference (Table 3). This amounts to an average read depth of 87.6×of the HCMV genome. Panels a in Fig. 3 show the coverage of all Nanopore reads aligned to the reference.

A comparison of the consensus sequence generated from the enriched HCMV reads aligned to the HCMV HHV-5 reference using the genomic similarity search tool YASS (Noé & Kucherov, 2005) revealed that the former had 99.4 % similarity to the reference (233 854 of 235 230 nucleotide residues). The conflicting/mismatch residues are mostly gaps in the Nanopore consensus sequence at positions 46 364–46 433 (proteins UL34 and UL35), 147 820–147 830 (helicase–primase subunit UL102), 194 363–194 698 and 195 851–195 977. The last two regions of difference coincide with inverted repeat regions (194 344–195 667, 195 090–197 626) (Masse *et al.*, 1992). A number of mismatches to the reference HHV-5 were identified upstream of base 1270; these were due to low coverage of this region by Nanopore reads. We found regions with low (<5×) coverage had a high number of mismatches compared with the reference, but areas of greater coverage matched near-perfectly.

**Fig. 4.**
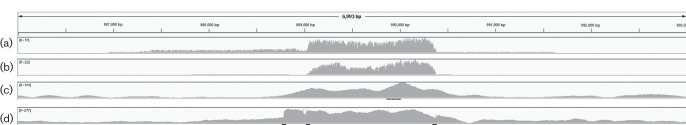
Coverage by Nanopore reads from DNA of *M. tb* H37Rv (a), *M. tb* strain C (b) and Illumina-sequenced long-fragment-enriched *M. tb* H37Rv (c) and *M. tb* strain C (d), shows a region (886 000–893 000) of high coverage in both Nanopore and Illumina experiments. Nanopore reads were aligned with BLASR, Illumina reads with Bowtie, visualised in IGV. Maximum read depth, as determined by IGV, is 17 (a), 22 (b), 331 (c) and 277 (d).

For *M. tb* strain H37Rv, we obtained a total of 2028 ‘pass’ and 9961 ‘fail’ reads (equivalent to 0.077×coverage) from four flowcells, for the strain *M. tb* C, 202 ‘pass’ and 46 711 ‘fail’ reads (0.182×) were obtained from three flowcells. Localized areas with high coverage were found in both strains; these were found to correspond to open-reading frames encoding transposases LH57_07500, LH57_18955, LH57_18175, and LH57_04320, at positions 887 429–887 488, 889 044–890 363, 1 538 580–1 539 822, 2 635 594–2 640 242, 3 544 391–3 547 252 and 3 788 312–3 789 669 of strain H37Rv (Fig. 4). These regions have been highly enriched compared with the background of human DNA, and also compared with the rest of the *M. tb* genome. The sample originally containing 10 % H37Rv DNA showed the highest rate of reads aligning to the reference, while both 90 % *M. tb* DNA samples (H37Rv and strain C) show enrichment mainly in the transposase regions.

### Sequencing of enriched long fragments on the Illumina MiSeq

To assess the success of the long fragment hybridisation, Illumina libraries were generated from the remaining half of the hybridised material, and sequenced on a MiSeq instrument (results shown in Table 4). A high percentage of influenza virus A and HCMV reads from long enriched fragments aligned to the target reference in both Illumina and Nanopore ‘pass’ reads.

Illumina-generated reads showed higher percentages of alignment than Nanopore reads, presumably due to the lower error rates. Illumina libraries generated from the hybridisation of long fragments, particularly the independently generated, 10 % *M. tb* H37Rv libraries 1–4 in Table 4, show successful enrichment of mycobacterial DNA, with 56–96 % of reads aligning to the H37Rv genome. Results for *M. tb* strain C show a relatively low rate of alignment of reads to the H37Rv genome in both Nanopore and Illumina experiments. This could be due to less successful enrichment, and an imperfect match of the *M. tb* strain C reads aligned to strain H37Rv, which has 98.9 % identity to a consensus sequence generated from our Illumina-sequenced *M. tb* strain C.

**Table 4.** Illumina MiSeq runs of independent hybridisations of long fragments The table shows the statistics of alignment generated by Bowtie (http://bowtie-bio.sourceforge.net/index.shtml) and coverage generated by Picard (http://broadinstitute.github.io/picard/).

Sample	Fragment hybridisation	Number of reads aligned to target pathogen	Percentage of reads aligned to target pathogen	Mean depth of pathogen coverage	Percentage of target bases covered at 10×
Influenza virus A	Long	2 664 967	59	2089	97
HCMV	Long	1 765 332	94	957	96
10 % *M. tb* H37Rv (1)	Long	6 906 339	84	295	99
10 % *M. tb* H37Rv (2)	Long	1 071 332	62	50	99
10 % *M. tb* H37Rv (3)	Long	8 193 188	96	315	99
10 % *M. tb* H37Rv (4)	Long	2 942 898	56	100	98
90 % *M. tb* H37Rv (5)	Long	2 258 868	93	99	99
90 % *M. tb* H37Rv (6)	Long	6 978 112	97	297	99
90 % *M. tb* H37Rv (7)	Long	9 926 437	87	342	99
90 % *M. tb* H37Rv (8)	Long	15 382 452	96	521	98
*M. tb* H37Rv (9)	Short	3 982 148	99	169	99
90 % *M. tb* strain C (1)	Long	689 141	18	24	85
*M. tb* strain C (2)	Short	2 980 023	99	115	97

Results from the enriched influenza virus A (Fig. S1c) show concordance with the coverage by Nanopore results (Fig. 2). The unevenness of the coverage is presumably a result of the prevalence of short fragments in the original RNA sample (Fig. S1a), reverse-transcribed to cDNA (Fig. S1b). Illumina reads (Fig. 3b) generally show less even coverage of the HCMV genome compared with Nanopore reads (Fig. 3a). Fifteen (out of a set of 23 525) aligned Nanopore reads span the repetitive replication origin oriLyt at position 94 488–94 588 (Chen *et al.*, 1999) (Fig. S2c, d). The complete (3.5 M aligned reads) Illumina dataset (Table 4) has a 100 bp gap in the alignment at this repetitive position (Fig. S2a, b). Two Nanopore reads cover the inverted repeat region 194 293–195 565, while no Illumina reads aligned in this gap, and almost all Illumina reads in the adjacent 2.5 kb region show a mapping quality equal to zero, when visualised in the IGV (Fig. S2g, h). A similar outcome has been observed for a comparison of Nanopore and 454 reads for human herpesvirus type 1 (Karamitros *et al.*, 2016). Areas with increased coverage can also be observed in Nanopore- and Illumina-generated datasets (Fig. 4) in *M. tb*. Here, this is presumably due to the redundancy of transposase-encoding sequences, which could result in localised increased aligning of reads.

## Discussion

This study explores the capture of specific, long DNA fragments for sequencing on a long-read platform, the Oxford Nanopore MinION™ instrument. We demonstrate that our method can be used to enrich large, specific regions of interest in mixed samples. Previous work by Greninger *et al.* (2015) has shown that detection of moderate to high titres of pathogen DNA (chikungunya virus, Ebola and hepatitis C virus) from human blood samples is possible using Nanopore sequencing. However, this direct sequencing approach is inefficient if the region of interest is a small subset of the total DNA, the target is of low titre, or if high coverage is required for strain typing and variant identification. In our Nanopore sequencing experiments with un-enriched influenza virus A and HCMV DNA (from cell cultures), we detected very low numbers of reads from the pathogen compared with those from the host cell line. In contrast, sequencing data from enriched DNA produced good coverage of the influenza virus A and HCMV genomes and partial coverage of the *M. tb* genome. Control experiments using Illumina sequencing to assess the quality of enrichment (Table 4, Fig. 4) showed good overall and minimum coverage, similar to the sample enriched by short-fragment hybridisation (Brown *et al.*, 2015; Christiansen *et al.*, 2014, sample 9 and *M. tb* strain C sample 2 in Table 4), indicating that the enrichment of long fragments does not introduce bias. Preferential enrichment of certain regions (Fig. 4) seems to be due to redundancy of the captured sequence, in this case the transposases.

The drawbacks of our method, compared with the high-throughput protocol used by Brown *et al.* (2015), and Christiansen *et al.* (2014), were lower target coverage and throughput. Enrichment and library preparation take approximately 28 h and include a 16 h hybridisation step and 3–4 h of long-range PCR. In the future, the enrichment step could be shortened to 4 h by using a different hybridisation protocol, and PCR amplification could be replaced with whole-genome amplification. Addition of molecular barcodes would allow pooling of several samples to be run simultaneously on one MinION™ flowcell. This, coupled with increasing speed, accuracy and throughput of MinION™ reads (e.g. results in Norris *et al.*, 2016), will reduce the time and number of reads necessary for strain and variant identification, making this method amenable for diagnostic purposes. The relatively inexpensive and small-footprint MinION™ sequencers have been used in settings where conventional Illumina sequencing would be difficult (Quick *et al.,* 2016).

We see the main application of our method of enriching long fragments in the detection of structural variants and in generating comprehensive coverage of specific target regions by long-read sequencing. Nanopore sequencing has previously been used to detect structural variants in pathogenic bacteria (Ashton *et al.*, 2014), human DNA samples (Ammar *et al.*, 2015) or human cancer cell lines (Norris *et al.*, 2016); we believe our method could be employed as a non-amplicon-based alternative for this application, improving library complexity and uniformity of the sample, and aiding the detection of single-nucleotide variants (Samorodnitsky *et al.*, 2015). As the enrichment approach is platform-agnostic, it could also be used to generate libraries compatible with the other long-read sequencers, benefitting the field of research into structural variation.

## Supplementary Data

2270 Supplementary File 1
