# Supplementary material for: Enrichment by hybridisation of long DNA fragments for Nanopore sequencing
Source: Microb Genom. 2016 Sep 20;2(9):e000087. doi: 10.1099/mgen.0.000087 (PMC5537632; doi:10.1099/mgen.0.000087)

Supplemental Figure S1: Agilent tape station visualization of RNA, cDNA and Illumina reads of the influenzavirus A sample. Panel **a:** input RNA (RNA tape) and **b:** cDNA (High sensitivity tape) shows distinct molecule sizes that are smaller than the influenzavirus A RNA fragments. Panel **c** shows Illumina MiSeq read distribution aligned to the influenzavirus A H1N1 reference with Bowtie. Maximum read depth for the fragments is: 4264 (NC_002023.1), 1845 (NC_002021.1), 6824 (NC_002022.1), 2891 (NC_002017.1), 12712 (NC_ 002019.1), 41552 (NC_002018.1), 9116 (NC_002016.1), 908 (NC_002020.1).

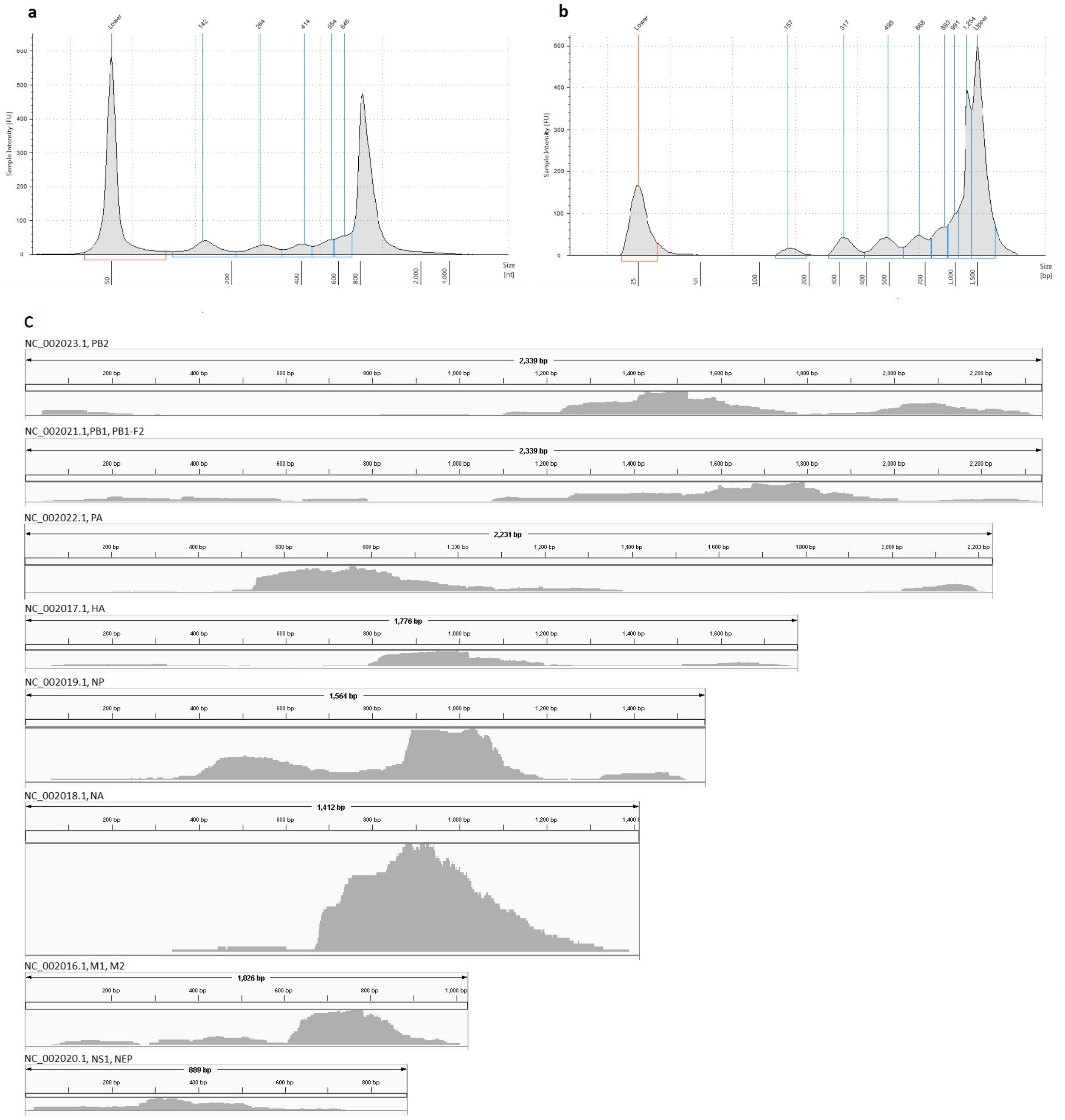

Supplemental Figure S2: HCMV regions of low coverage and read pileups, visualised in IGV. Panels **a-d** show OriLyt; Illumina coverage plot (**a**, maximum: 1008) and Bowtie-aligned read pileup (**b**), and Nanopore coverage plot (**c**, max. 16) and pileup (BLASR alignment, **d**). Panels **e-h** show the repetitive region at position 195000; Illumina coverage (**e**, max. 5438) and pileup (**f**), and Nanopore coverage (**g**, max. 83) and pileup (**h**) are shown. White reads in panel f indicate a mapping quality close to zero, here due to the redundancy of the sequence, that prevents the unambiguous mapping of short reads.

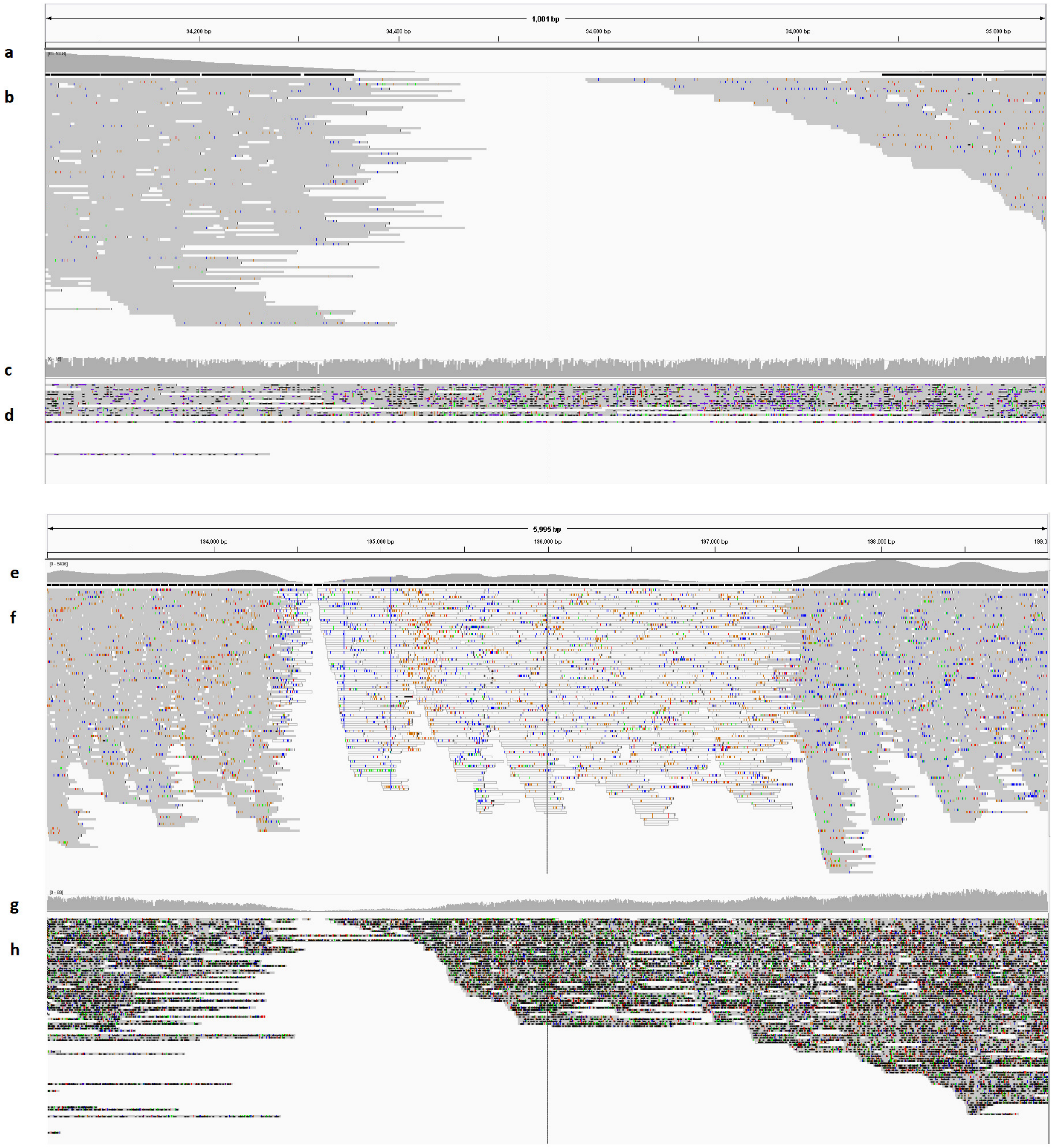

Supplement: Supplementary File 1 [file mgen-02-87-s001.pdf]
